# Single-shot frequency offset measurement with HASTE using the selective parity approach

**DOI:** 10.1038/s41598-024-60275-4

**Published:** 2024-04-30

**Authors:** Irina de Alba Alvarez, Aidin Arbabi, Vitaliy Khlebnikov, José P. Marques, David G. Norris

**Affiliations:** 1 Donders Institute for Brain, Cognition and Behaviour, Centre for Cognitive Neuroimaging, Radboud University, Nijmegen, The Netherlands; 2 Erwin L. Hahn Institute for Magnetic Resonance Imaging UNESCO World Cultural Heritage Zollverein, Kokereiallee 7, Building C84, 45141 Essen, Germany; 3 Department of Clinical Neurophysiology (CNPH), Faculty Science and Technology, University of Twente, Enschede, The Netherlands; 4 Multi-Modality Medical Imaging (M3I), Faculty of Science and Technology, University of Twente, Enschede, Netherlands

**Keywords:** Biophysics, Medical imaging

## Abstract

Measurements of frequency offset are commonly required in MRI. The standard method measures the signal phase as a function of evolution time. Here we use a single shot turbo-spin-echo acquisition method to measure frequency offset at a single evolution time. After excitation the transverse magnetisation evolves during the evolution time, and is then repeatedly refocused. The phase is conjugated between alternate echoes. Using partial parallel acquisition techniques we obtain separate odd- and even- echo images. An iterative procedure ensures self-consistency between them. The difference in phase between the two images yields frequency offset maps. The technique was implemented at 3 Tesla and tested on a healthy human volunteer for a range of evolution times between 6 and 42 ms. A standard method using a similar readout train and multiple evolution times was used as a gold-standard measure. In a statistical comparison with the gold standard no evidence for bias or offset was found. There was no systematic variation in precision or accuracy as a function of evolution time. We conclude that the presented approach represents a viable method for the rapid generation of frequency offset maps with a high image quality and minimal distortion.

## Introduction

**Figure 1:**
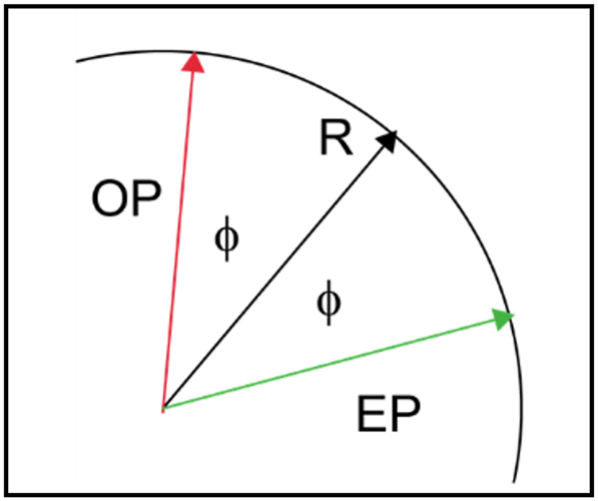
Schematic showing how the odd- and even-parity echoes are bisected by the reference phase in a CP sequence for any arbitrary voxel. The assignment of OP/EP is arbitrary. The orientation of R is determined by the sensitivity profile of the receiver coil relative to the transmit field and will vary for any given voxel and receiver coil.

Frequency offset (FO) maps are commonly required in MRI, for example to correct for distortion in echo planar imaging (EPI) experiments^1^, or to calculate temperature changes using changes in proton resonance frequency during hyperthermia^2,3^. The standard approach to obtaining such maps is to acquire data at multiple evolution times (τ) and to estimate FO from the phase evolution. This is typically done using a multi-echo gradient-echo approach, which then allows the calculation of both T_2_^*^ and FO. To estimate T_2_^*^ it is necessary to obtain data at multiple evolution times and fit the acquired data to a signal decay model. In theory the FO could be calculated from a single acquisition if the absolute phase (relative to that at zero evolution time) would be known rather than an arbitrary phase value. In the present contribution we examine the application of a Carr–Purcell (CP) echo train to obtain two images with equal but opposite phase offsets relative to a reference phase, which we define as the phase the magnetisation would have if it would satisfy the Carr–Purcell–Meiboom–Gill (CPMG) condition. We have chosen to explore this approach using the single-shot half-Fourier acquisition single-shot turbo spin echo imaging (HASTE) method, in which an additional evolution period is inserted between the excitation and first refocusing pulse. In the presence of a frequency offset the magnetisation will evolve by some angle (φ) away from the reference angle. Depending upon the number of refocusing pulses experienced the magnetisation will be oriented at (+φ) or (−φ) corresponding to signals with odd or even echo parities (termed OP, EP in shorthand) as illustrated in Fig. 1.

Although the potential to utilise the phase evolution, for example in spectroscopic imaging, was realised early in the development of non-CPMG turbo spin-echo (TSE)^4–6^ it has been more common to use this sequence to generate undistorted T_2_^*^-weighted images^4–7^. In order to obtain a single shot FO measurement, we need to reconstruct OP and EP images separately. Consequently, not all solutions to the non-CPMG TSE problem can be used, because methods that eliminate one echo parity are intrinsically unsuitable. Possible approaches include split acquisition of fast spin-echo (SPLICE)^8^, 2 in 1 rapid acquisition with relaxation enhancement (RARE)^9^, the methods proposed by Gibbons et al.^10,11^ and the selective parity approach^12^. In SPLICE the readout gradient is doubled in duration to allow OP and EP echoes to be acquired separately. Dummy cycles are required so that the amplitudes of both are similar. The data are reconstructed separately and combined to form a final magnitude image. In 2 in 1 RARE spin and stimulated echoes are generated at the start of an echo train and refocussed separately, allowing the interleaving of two separate contrasts. In the approach of Gibbons et al.^10,11^, a little more than half of the k-space is acquired with alternating odd and even parity echoes, which are then reconstructed separately using a partial Fourier approach. The selective parity method acquires either an OP or an EP line at each echo, and in the original approach combines them to form a single image. In the present contribution we instead use partial parallel reconstruction methods to generate separate OP and EP images. In contrast to the methods of Gibbons et al.^10,11^, we acquire the whole of k-space, and do not require dummy cycles. The differences between the methods mentioned here is relatively minor, and we expect the approach we follow here to be readily translatable to other non-CPMG TSE techniques. We note parenthetically that single shot RARE methods have also been used to map temperature changes on the basis of changes in T_2_^13^.

## Materials and methods

**Figure 2:**
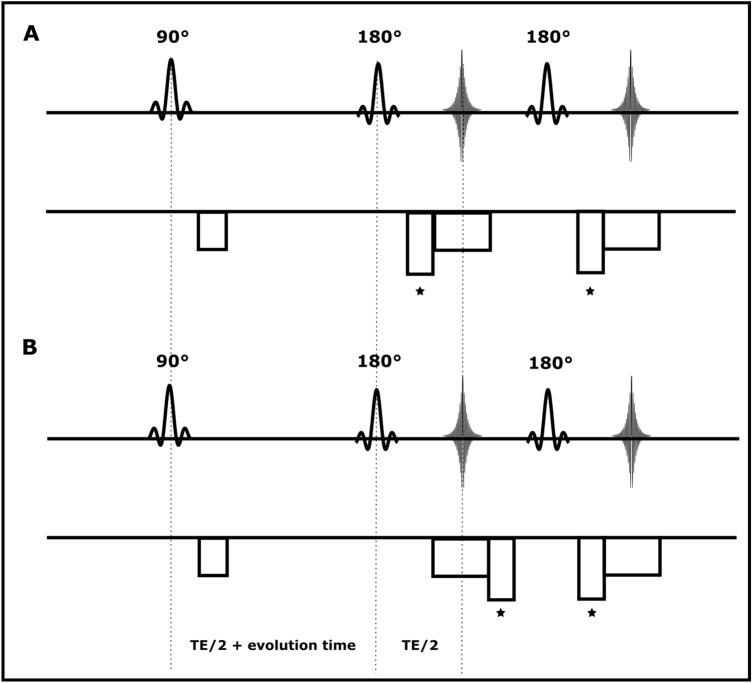
The timing diagram of (**A**) HASTE, (**B**) SP-HASTE methods: a variable evolution time was implemented between the excitation and the first refocusing RF pulses. Displacing gradients, as depicted by asterisks, were used to acquire the parity of interest and avoid destructive interference between parities. Only the RF pulse train and frequency encoding direction for the first 2 echoes are shown. Dummy echoes are not shown for HASTE timing diagram in (**A**).

The original Siemens diffusion-weighted HASTE pulse sequence as a variant of the HASTE method was modified through implementing a variable evolution time between the 90° excitation and the first 180° refocusing RF pulses, and removing the diffusion encoding preparation module. Although this sequence is termed ‘HASTE’ it uses centre-out phase-encoding, and the readout is as described in Norris et al^4^. To avoid artefacts arising from the non-CPMG condition due to the evolution of spins during the evolution time, a displacing gradient was used prior to each read-out gradient to remove the odd parity echo out of the acquisition window, and only the even parity echo was acquired at each echo time (Fig. 2A). For stabilisation of the early echo amplitudes, the use of a minimum of four dummy cycles was necessary before the readout train (not shown in Fig. 2A). This sequence was used to obtain a gold standard measurement using the classical approach of incrementing the evolution time between the excitation pulse and the readout, and calculating the frequency offset as the slope of the phase as a function of τ. These data could then be compared to those obtained by the selective parity (SP) method.

The main experiment was implemented using the SP-HASTE method^12^. The pulse sequence was slightly modified compared to HASTE by applying displacement gradients either before or after the read-out gradient to acquire the echo parities in an alternating fashion (Fig. 2B), and by no longer applying dummy cycles. A phase graph showing how different coherence pathways combine to form the odd and even parity echoes is shown in Fig. 2 of Norris^12^.

**Figure 3:**
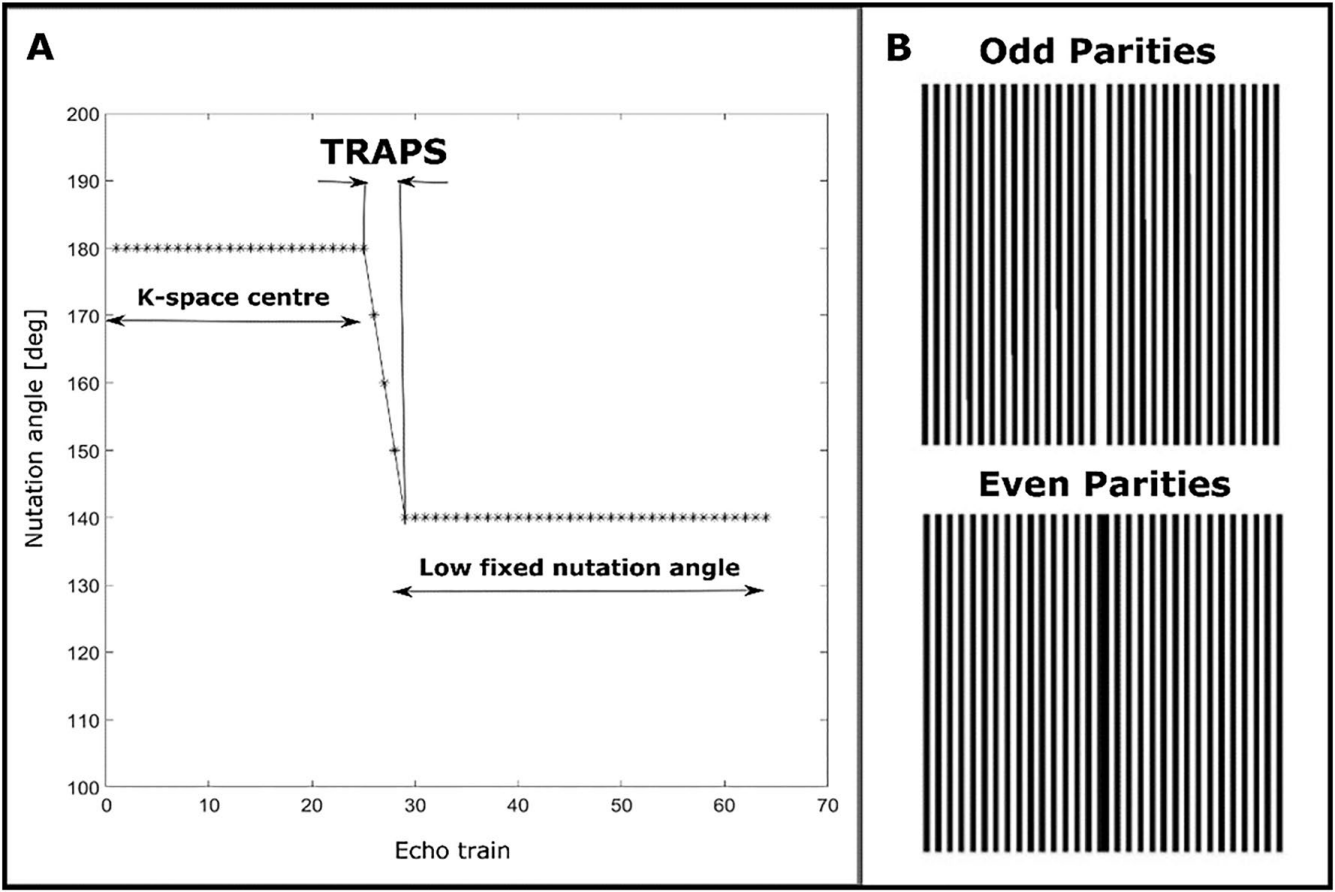
SP-HASTE: (**A**) TRAPS to reduce the SAR contribution from SLR refocusing pulses. The k-space centre is sampled using nominally 180° pulses, and then, the nutation angle is reduced to a fixed low flip angle of 140°. (**B**) The centre-out phase encoding scheme to sample the k-space while alternating echo parities, starting with the odd echo parity for the k-space central line (white indicates acquired data).

In the SP-HASTE method, Shinnar le Roux refocusing RF pulses^14^ replaced the original truncated sinc refocusing RF pulses. Their superior slice profiles result in a smooth signal intensity decay over early echoes. This makes the use of dummy cycles for the echo amplitude stabilisation unnecessary leading to a shorter echo time and higher signal intensity. Better slice profiles of the SLR RF pulses come at the cost of a higher specific absorption rate (SAR) contribution, which was compensated by using a smooth transition between pseudo steady states (TRAPS^15,16^, Fig. 3A) for the later echoes. A centre-out phase encoding scheme was also employed to sample the k-space with evenly distributed odd and even echo parities (Fig. 3B).

MRI scans were performed on a whole body 3T Siemens Prisma/PrismaFit scanner (80 mT/m strength and 200 mT/m/ms slew rate) with a 32-channel receive-only head coil in one healthy female volunteer. Ethical approval was provided by the local ethics committee METC Oost-Nederland, which is an accredited Dutch ethical reviewing board. The approval is registered under CMO2014/288 entitled ‘Imaging Human Cognition’. Informed written consent was obtained. All experiments were performed in accordance with the relevant guidelines and regulations. Common acquisition parameters were as follows: TR = 2000 ms , FOV = 200 × 200 mm, matrix size of 64 × 64, 3.1 mm isotropic in-plane resolution, three axially oriented 3 mm slices, and 20 repetitions for the SP-HASTE method. No accelerated imaging was used for the data collection. Data were collected with evolution times ranging from 0 to 42 ms with increments of 6 ms. TE was 34.65 ms and 6.93 ms for the HASTE and SP-HASTE, respectively. The echo spacing was 6.93 ms for both sequences. The longer TE was necessitated by the presence of the four dummy cycles.

### Reconstruction

**Figure 4:**
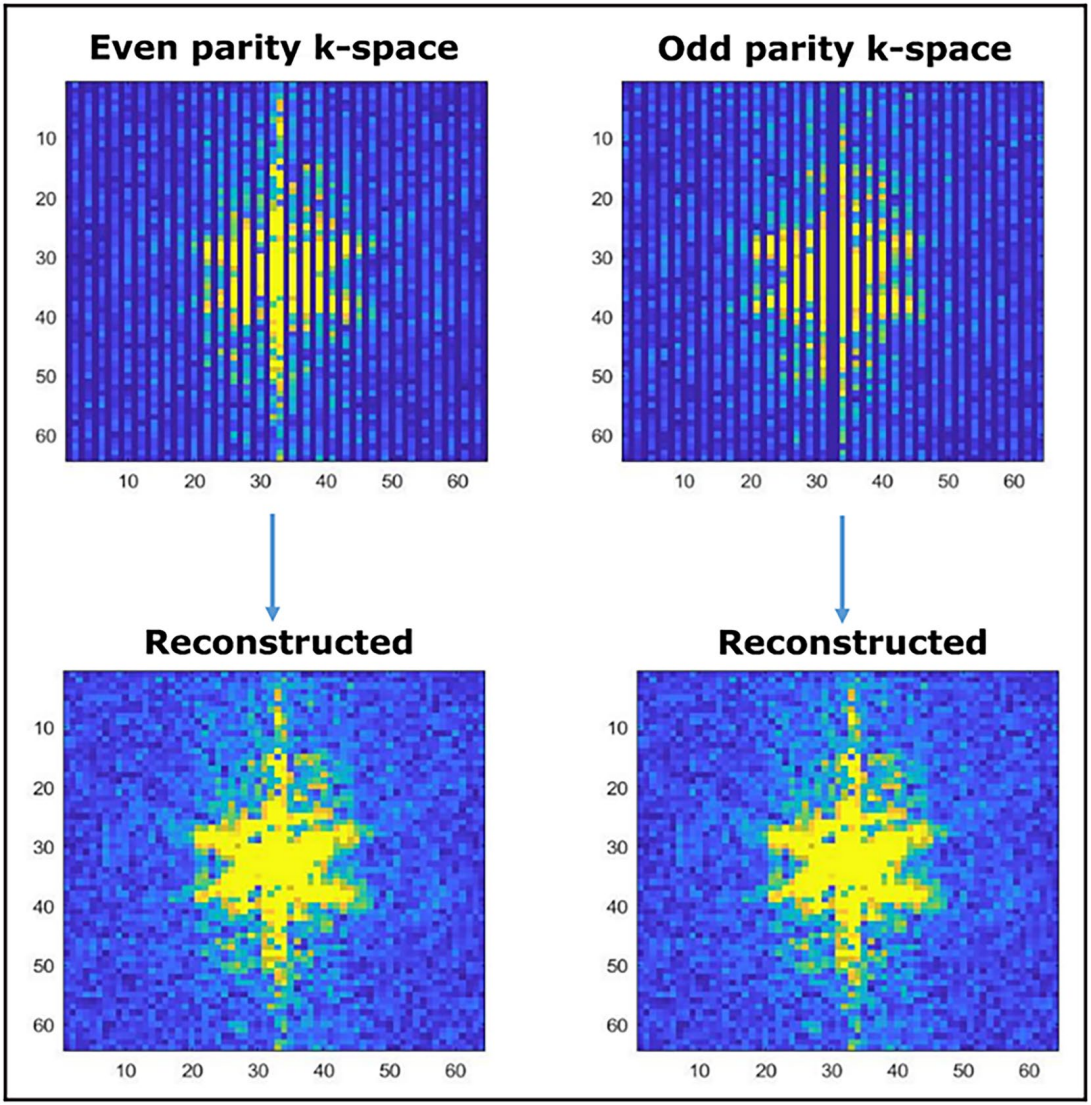
Even and odd echo parity k-spaces created in the reconstruction: undersampled lines can be seen in the original k-spaces (top row). The bottom row shows the odd and even parity k-spaces with the missing lines estimated through the SPIRiT algorithm.

The HASTE data were reconstructed using the standard reconstruction algorithm delivered by the manufacturer. A novel reconstruction approach was used for the SP-HASTE data to allow separate reconstruction of odd and even parity images using in-house code written in matlab (MATLAB and Statistics Toolbox Release 2012b, The MathWorks, Inc., Natick, Massachusetts, United States). The acquired echo parities were used to create two separate k-spaces (Fig. 4), one for each parity. The odd and even k-space missing lines were then estimated through SPIRiT (GRAPPA) algorithm^17^.

**Figure 5:**
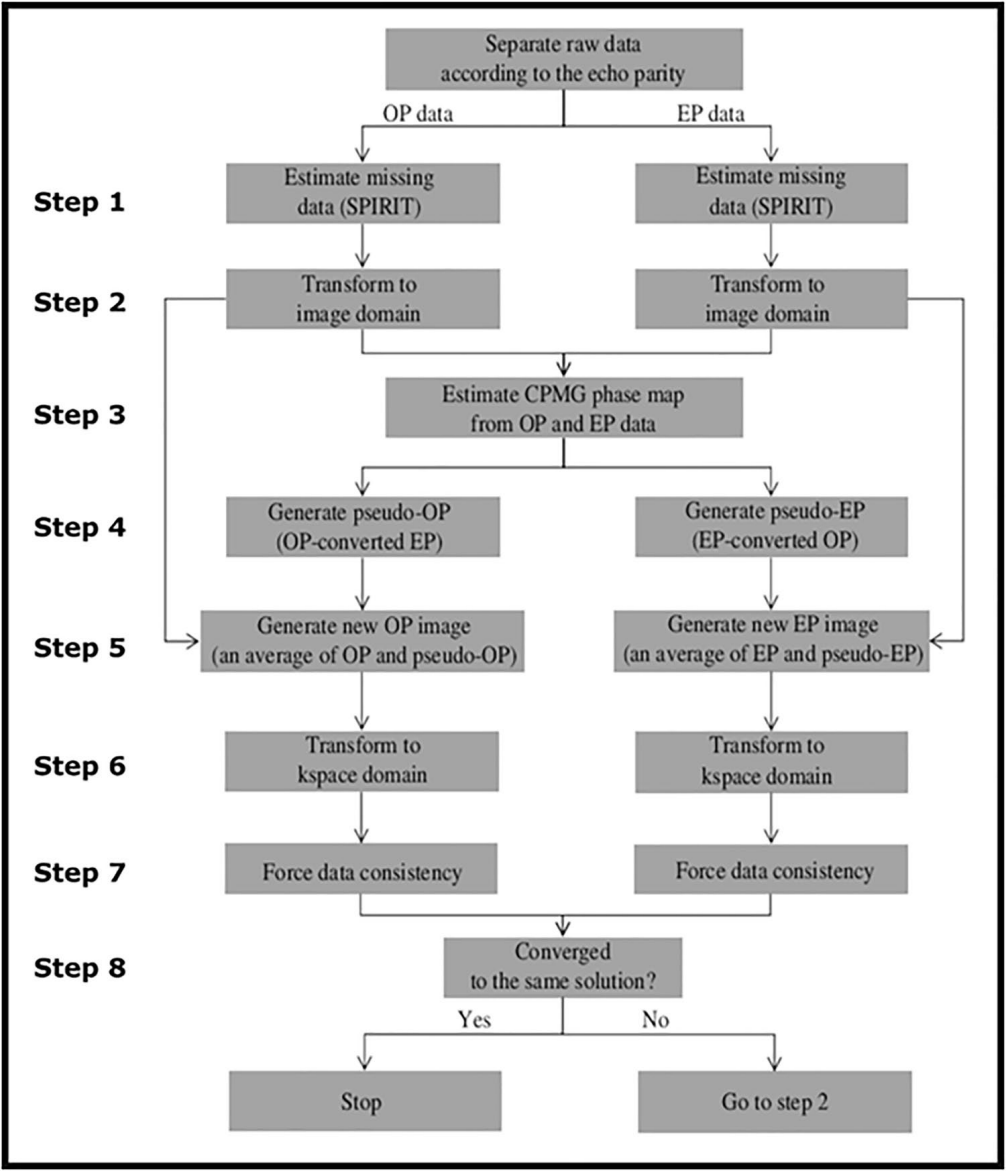
Schematic of the SP-HASTE reconstruction performed for each receiver element. The OP and EP data are separated and missing k-space lines are filled using SPIRiT (step 1). Transformation into the image domain (step 2) results in two images that should have a complex conjugate relationship defined relative to the CPMG angle. An estimate of the CPMG angle is obtained as the angle bisecting the OP and EP phases in each voxel (step 3). Using this estimate of the CPMG phase angle it is then possible to transform OP data to EP data and vice versa generating pseudo OP and EP data (step 4). The original and pseudo data are then averaged (step 5) and transformed back into k-space (step 6). Data consistency is enforced by restoring the originally acquired data for each parity (step 7). If the resulting magnitude images are identical to within 1% tolerance the procedure is terminated (step 8) or a new iteration is performed.

RF single loop receiver coils have an inhomogeneous reception field and a spatially varying receiver field with an arbitrary phase relationship to the orientation of the transmit field. By reference to Fig. 1, the odd and even parity images are complex conjugates of each other in the image domain considering the CPMG angle as the frame of reference. An iterative process was then used to ensure that the final images satisfied this condition. This iterative approach converges to a consistent representation of the non-acquired data lines as is depicted in Fig. 5. The critical step is the estimation of the CPMG phase map (step 3), which is subsequently used to convert odd parity to even parity data and vice versa (step 4). The information from both echo parities is then combined in step 5. After ~ 30 iterations (relative error below 1%), odd parity and even parity images eventually converge to the same solution, providing identical magnitude images. The final magnitude images were generated using the sum of squares of the coil images. For frequency offset (FO) mapping, SEPIA, a Quantitative Susceptibility Map (QSM) software^18^ was used to unwrap the phase data using the SEGUE method^19^, and generate the phase images for both the Siemens and the selective parity data.

## Results

For the modified SP-HASTE sequence, the 0 ms delay time was disregarded since no phase shift is generated between the odd and even parity echoes. The FO maps were generated using Eq. (1):

1 $$FO=\frac{{\phi }_{e}-{\phi }_{o}}{2\tau }$$

where ɸ_e_ and ɸ_o_ are the even and odd parity phase images, respectively, and τ is the evolution time.

**Figure 6:**
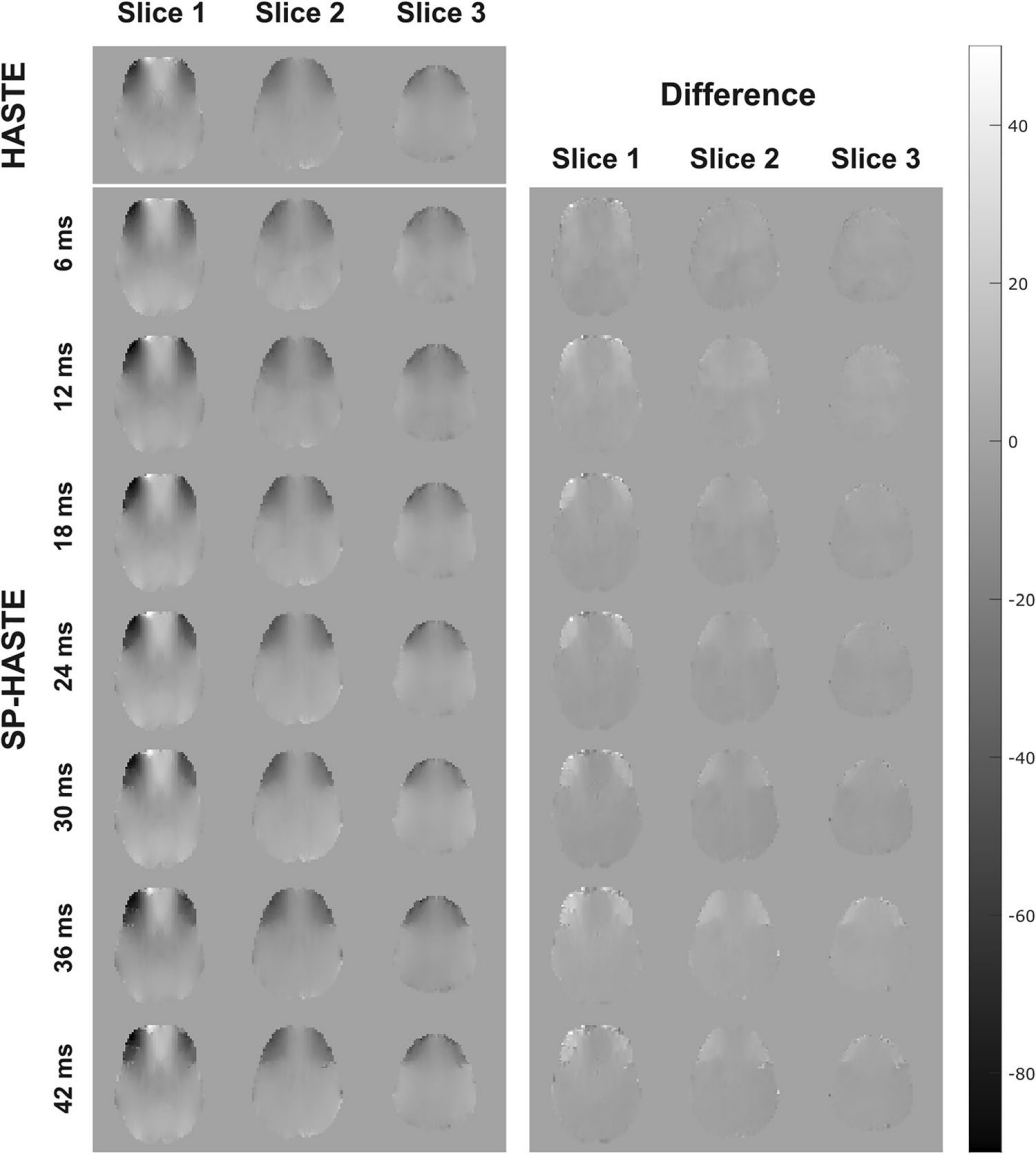
Left, frequency offset maps created with the HASTE (top) and single shot SP-HASTE (2nd row down) methods: FO maps from the SP-HASTE methods were calculated for different evolution times from one repetition. Grey scale units are expressed in Hz. In the right column the difference between the two is shown (HASTE-SP-HASTE).

The FO maps created with HASTE and from single shot SP-HASTE acquisitions at different evolution times are presented in Fig. 6.

**Figure 7:**
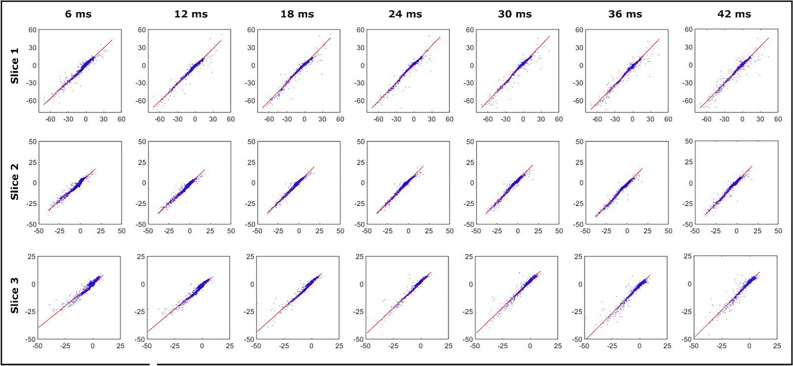
Linear regression of the FO values obtained with the SP-HASTE (vertical axes) versus those obtained with HASTE (horizontal axes) for the same sample axial slices as in Fig. 6 (top to bottom) for different evolution times (left to right). The red solid line on each panel represents the calculated slope. The blue dots are the individual data points (voxels within slice). Each data point for SP-HASTE represents the mean over 20 repetitions. Units are expressed in Hz.

By collecting multiple repetitions of the SP-HASTE we can explore whether there is a systematic difference in the FO values recorded when taking HASTE as a gold-standard measure, as averaging over the repetitions should ensure sufficient SNR that systematic errors become visible. To this end a linear regression was performed for the FO mean values obtained with the SP-HASTE for all evolution times versus the FO mean value from the HASTE method. For the SP-HASTE method, FO values were averaged over 20 repetitions. Table 1 shows that differences in the slope between the two techniques were less than 6% for all evolution times (0.94 < slope < 1.06) and that the offset was in all cases less than 3 Hz. The corresponding data are shown in Fig. 7.

**Table 1:** Slope and intercept values as a function of evolution time.

Evolution times (ms)	6	12	18	24	30	36	42
Slope	0.9497	0.9912	1.0321	1.0447	1.0522	1.0327	1.0246
Intercept (Hz)	0.2317	− 1.9286	0.6326	1.2022	1.9472	− 2.5518	0.0891

The predicted linear regression slope and intercept for slice 1: linear regression was performed for the FO mean values obtained with SP-HASTE for evolution times ranging from 6 to 42 ms vs. the FO mean value from the HASTE.

**Figure 8:**
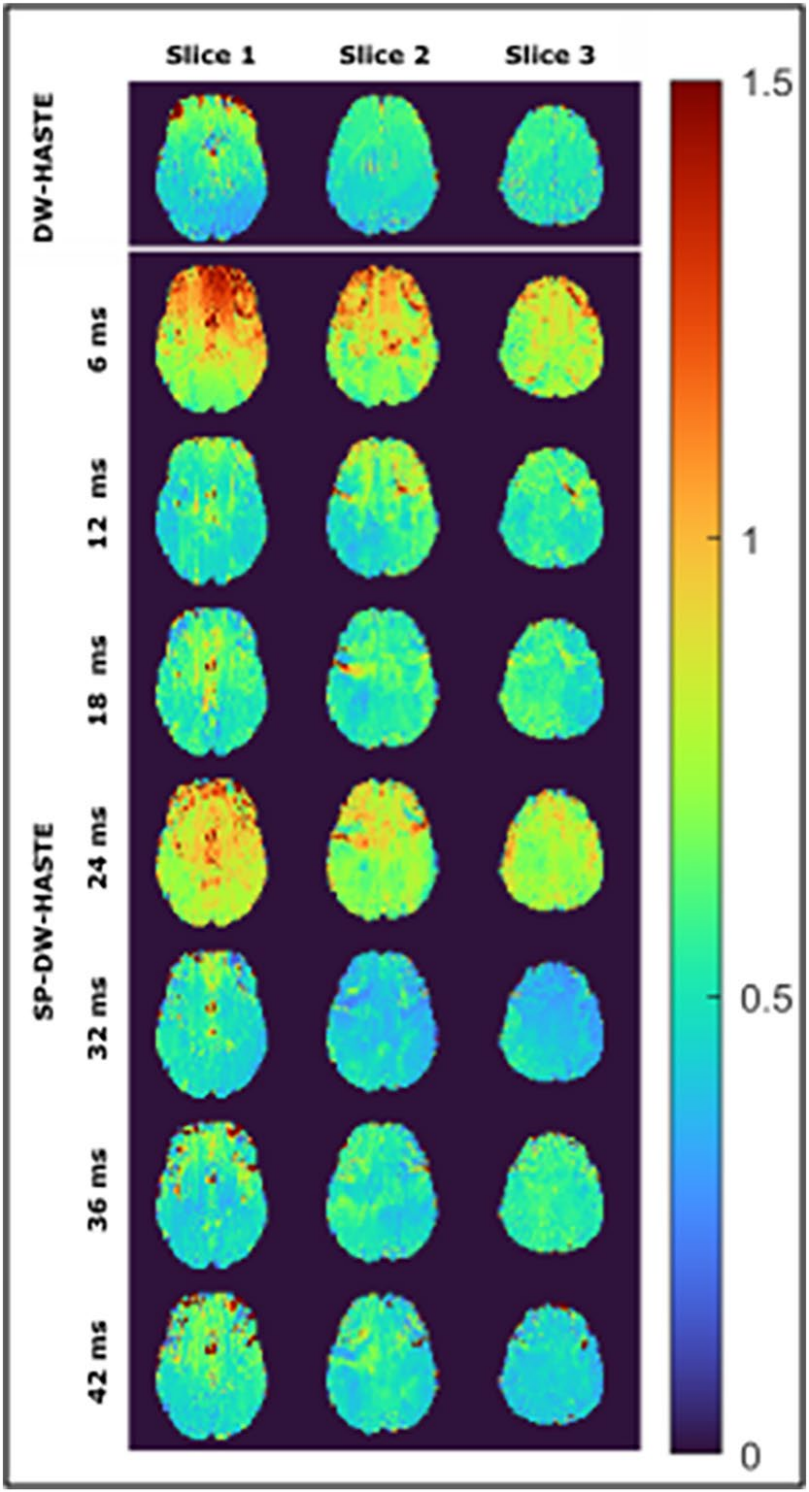
Temporal standard deviation maps (in Hz) calculated over 20 repetitions for the FO values obtained with the proposed methods for different evolution times: The SP-HASTE FO maps obtained with 6 and 24 ms evolution times show a higher Std compared to the HASTE method. The SP-HASTE FO map estimated for 32 ms delay time has smaller Std compared to the HASTE method for slices 2 and 3. A comparable agreement can be observed between the two methods for the other evolution times.

For the HASTE measurements the standard deviation could be calculated from the measured slope and for the SP-HASTE it could be obtained from the variance over the repetitions. Calculated Std maps are presented in Fig. 8 for the FO maps obtained with the HASTE and SP-HASTE. Overall, similar standard deviation values are observed. Slightly higher Std values are observable for the SP-HASTE FO maps obtained from 6 to 24 ms evolution times which reflect more fluctuation between the measurements for these evolution times.

**Figure 9:**
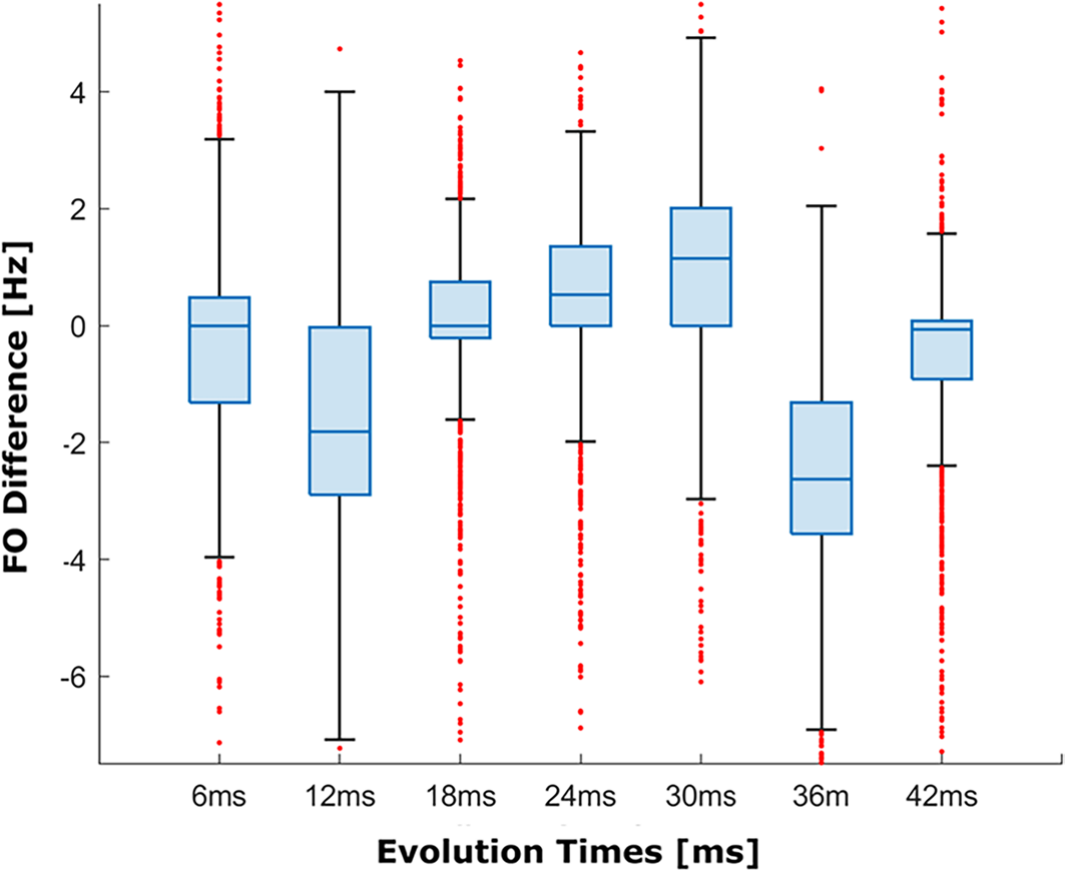
Box plot of the difference between the FO values estimated using the SP-HASTE and HASTE for slice 1 as shown in Fig. 6 for all the evolution times, calculated on a pixel by pixel basis.

Figure 9 illustrates the difference between the FO values estimated with the two methods for all the evolution times for slice 1. The smallest median difference was found for 6 ms and 18 ms evolution times, with the FO difference close to 0 Hz. The largest median difference was for 36 ms delay time with a median difference of 2.62 Hz. The evolution times which showed the narrowest whiskers were 18 ms and 42 ms.

## Discussion

In this paper we have demonstrated that a single shot non-CPMG HASTE sequence that acquires both odd and even parities of echoes is capable of generating field maps with the same precision and accuracy as the slower standard method. This capability could have application in generating undistorted FO maps for EPI distortion correction, and for monitoring temperature changes, for example in hyperthermia. This approach relies on obtaining separate images corresponding to the two parities of echo, and relies on the complex conjugate relationship between the echo parities at the voxel level. CP sequences that acquire the two parities in an alternating fashion are an obvious choice for such an experiment as demonstrated herein, but a two echo asymmetrical spin-echo EPI experiment could also be used to generate similar maps. If it is not possible or desirable to use parallel imaging techniques to generate separate OP and EP images then they could be obtained in two separate acquisitions. The resultant FO map would be generated under the assumption that the phase was stable between the two scans. It would also be necessary to insert dummy scans as in the standard method to avoid fluctuations in the echo intensity^4^. An alternative two-shot approach that would be less sensitive to motion-induced phase variation would be to collect a reference phase map using a CPMG sequence (evolution time of zero) and compare this with the phase of an OP/EP acquisition.

One slight surprise in the results obtained was the relative insensitivity of the FO maps to the additional delay. Increasing the delay time will increase the phase evolution which will be a linear effect, while reducing the SNR, which will decay with T_2_^*^. We chose the range of evolution times to have a good coverage up to and beyond the anticipated T_2_^*^, but it would seem that at 3 T a delay of 6 ms is already sufficient to generate high quality FO maps. The rapid advance of denoising techniques^20^ that reduce the thermal noise contribution can potentially reduce the minimum usable delay still further.

In this work we examined the phase evolution under the influence of static magnetic field inhomogeneities in the brain at 3 T. Different organs, higher static magnetic field strengths, or the presence of metallic implants would all present a greater challenge. If the frequency offset would be caused by chemical shift then the frequency could change abruptly with spatial position, and the maximum phase difference that could be allowed to evolve should not exceed π. If the frequency offsets are caused by susceptibility differences then there will be a continuous gradient in the frequency offset, and within the constraints of phase-unwrapping algorithms, this can be unwrapped and phase evolutions > π within a voxel corrected.

In comparison with the commonly used multi-echo gradient-echo approach the current method has the advantage that it does not conflate the imaging parameters with the evolution of the magnetisation. In the multi-echo approach a large frequency offset could require a short inter-echo spacing and conversely measurement of small frequency offsets can prolong the echo train and increase the acquisition time. One further advantage of the current method is that there is no minimum TE, so in the presence of strong inhomogeneities there is no minimum evolution time: if signal can be excited then its frequency offset can be measured. The present approach will work less well for tissue with short T2 and will also have higher RF power deposition. It could prove particularly attractive for applications at low static magnetic field where field homogeneity is poor.

In conclusion, we have demonstrated a novel approach for mapping frequency offset that removes the requirement to measure at multiple evolution times, and offers the prospect of accurate single shot measurement with high image quality and negligible distortion.

## Data Availability

The datasets used and/or analysed during the current study are available from the corresponding author on reasonable request.
